# Tackling Methodological Challenges in Nursing Home Research

**DOI:** 10.1093/geroni/igaf122.451

**Published:** 2025-12-31

**Authors:** Susan Hickman, Andrew Zullo, Julie Lima, Cathleen Colon-Emeric, Jennifer Stevens-Lapsley, Stefan Gravenstein, Melissa Tran

**Affiliations:** Indiana University, Indianapolis, Indiana, United States; Brown University, Providence, Rhode Island, United States; Brown University School of Public Health Department of Health Services, Policy, and Practice, Providence, Rhode Island, United States; Duke University School of Medicine, Durham, North Carolina, United States; University of Colorado Anschutz Medical Campus, Aurora, Colorado, United States; Alpert Medical School of Brown University, Providence, Rhode Island, United States; University of Colorado Anschutz Medical Campus, Aurora, Colorado, United States

## Abstract

The NEXT STEPs Methods, Measures, and Data Core (MMDC) develops guidance for researchers conducting studies in nursing homes through consultation and best practice recommendations. To help inform Year 1 priorities, a survey administered to a convenience sample of early-stage investigators (n = 23) identified key challenges, including regulatory and institutional review board (IRB) considerations, institutional engagement and resistance, and study design concerns. Concurrent with the survey and then drawing on its responses, the MMDC synthesized best practices through literature review, regulatory analysis, and expert consensus. Key guidance focuses on: 1) research regulation and compliance, including navigating HIPAA requirements and when to execute Business Associates Agreements, Data Use Agreements, Research Services Agreements, and Federal Wide Assurances; 2) accessing and using primary and secondary data sources relevant to nursing home research; and 3) selecting appropriate clinical trial designs in the nursing setting as well as strategies to overcome barriers to research. Conducting research in nursing homes presents unique challenges related to developing partnerships, identifying data sources, and study design. Newly created tools and resources, including a data sources compendium and best practice IRB and regulatory compliance guidelines, are available from the NEXT STEPS MMDC to help investigators navigate these complexities and grow the evidence base of nursing home explanatory clinical trials.

